# Food Values

**Published:** 1874-11

**Authors:** 


### FOOD VALUES.

All the food we eat yields to the body either warmth or strength; most articles give both; some warmth only, and not a particle of strength. Among these are "drippings," sweet oil, lard, butter, sugar and molasses; hence these things are used more freely on our tables in winter, when we need warmth, than in summer, when the great desire is to "keep cool."

The elements of food which give warmth and strength are carbon and nitrogen, which being taken into the system form fat and flesh; the fat, being consumed, makes the body a stove; the flesh, on the other hand, represents strength, or the muscles; hence when a muscular man is spoken of, it is understood that he is a strong man.

The scientific analysis of food demonstrates that various kinds give various degrees of warmth and strength. A quart or pound of beer gives but one grain of strength; a pound of bread ninety grains, a pound of beef one hundred and seventy-five grains, while a pound of cheese made out of skimmed milk yields three hundred and sixty-four grains of strength; whereas a pound of fresh butter gives 4,712 grains of warmth, but not an atom of strength.

In *Health by Good Living*, which has reached its twenty-first edition in three years, is a table containing the nutritive value of various kinds of food, from which the following is taken, giving the amount of strength in grains which a pound of each article named affords the system after being taken into the stomach:—

| One Pound of | Yields grains in Strength: |
|---|---|
| Beer or Porter | 1 |
| Parsnips | 12 |
| Turnips | 13 |
| Whey | 13 |
| Carrots | 14 |
| Greens | 14 |
| Potatoes | 24 |
| Skimmed milk | 34 |
| New milk | 35 |
| Buttermilk | 35 |
| Barley meal | 70 |
| Rice | 70 |
| Green bacon | 79 |
| Rye meal | 88 |
| Bakers' Bread | 90 |
| Pearl barley | 91 |
| Dry bacon | 98 |
| Fresh pork | 108 |
| Seconds flour | 120 |
| Indian meal | 123 |
| White fish | 130 |
| Cocoa | 130 |
| Oatmeal | 140 |
| Mutton | 140 |
| Beef | 175 |
| Bullock's liver | 210 |
| Red herring | 217 |
| Split peas | 255 |
| Chedder cheese | 315 |
| Skim cheese | 364 |

If this table was placed in the hands of the intelligent poor who have to work for a living, it would be a greater charity than to give them money, because it would lead them to purchase articles of food which give the most strength to work; and yet it is pitiful to think what multitudes, who have to work hard and early and late to earn every cent they get, thoughtlessly spend ten cents for a quart of beer which yields only one grain of strength, whereas if spent for a loaf of bread, it would give seventy times the ability to work.

There are many places in the country where a pound of cheese made out of skimmed milk can be purchased at retail for ten cents, yielding three hundred and sixty-four times more strength to labor than the same amount laid out in common beer.

The Germans in our country are the most frugal of our population, and the most thrifty.

"How is it that your cobbler repairs shoes at such a low price?" said a gentleman one day to his merchant neighbor.

"Oh, the Dutch live on liver."

If the reader will turn to the table, he will see that a pound of beef liver gives nearly one-third more strength to labor than a pound of beef costing more than double. In Cincinnati millions of gallons of buttermilk are sold every year at one half the price of sweet milk—the Germans are almost exclusively the purchasers—and yet the table shows that it gives as much strength as an equal amount of sweet milk. In a physiological direction, sweet milk causes biliousness, while buttermilk, by its acidity, cures it.

SUNDAY EATING.

As Sunday is a day of rest, and does not require such a putting out of strength as on other days of the week, a wise economy would dictate the purchase of the cheaper kinds of food which are less productive of strength, such as spinnage, turnips, carrots, parsnips and the like, costing but a cent or two a pound, instead of making the Sunday dinner the best of the week, with beef and mutton and poultry, costing ten times as much.

WARMING FOOD.

If we know what kind of food imparts most warmth, and what least, we could intelligently adapt the food to the weather, as we adapt our fires and our clothing to the season of the year. Buckwheat cakes, with butter and molasses, make a very common winter morning's breakfast dish, and each of the three of the ingredients is almost entirely carbonaceous, warmth producing. Our instincts lead us to avoid these in midsummer, and to select fruits and berries, which yield little or no warmth. It would be as much out of place to eat these in July as it would be to build a blazing fire on the hearth, or to wear woolen tipped with fur in the dog days. If, then, a man understands the value of food in its relation to warmth, he may wisely adapt it to the season of the year to great advantage in cases where he expects to have to encounter protracted cold or be exposed to summer heats. With this view the following table is arranged so that it can be seen at a glance what is the relative value of food as to its capacity of warming the system:—

| One Pound of | Yields grains of Warmth: |
|---|---|
| Whey | 154 |
| Turnips | 238 |
| Beer and porter | 315 |
| Buttermilk | 335 |
| Skimmed milk | 350 |
| New milk | 378 |
| Carrots | 385 |
| Spinnage | 420 |
| Parsnips | 421 |
| Potatoes | 770 |
| White fish | 900 |
| Beef liver | 1226 |
| Red herrings | 1435 |
| Bakers' bread | 2000 |
| Molasses | 2200 |
| Beef | 2300 |
| Skim cheese | 2350 |
| Chedder cheese | 2500 |
| Seconds flour | 2650 |
| Rye meal | 2650 |
| Rice | 2730 |
| Split peas | 2730 |
| Barley meal | 2750 |
| Indian meal | 2800 |
| Oatmeal | 2800 |
| Sugar | 2800 |
| Fresh pork | 3000 |
| Green bacon | 4000 |
| Dry bacon | 4300 |
| Fresh butter | 4700 |
| Suet | 4700 |
| Lard | 4800 |
| Drippings | 5500 |

In the almost wholesale use of lard, suet, fresh pork and buckwheat cakes in winter, there is an illustration of the wisdom of the habits and instincts of the people in connection with their eating and drinking; science corroborating instinct and custom, as also in the general fact that we consume less meat and more vegetables and fruit in summer time than in winter. At the same time, if a man is in perfect health, he should not eat by rule and measure, and order his table by square and compass, but wisely eat that which his appetite craves, what he likes best, and thus enjoy himself to the full without being hampered by rules and regulations, knowing that all things good to eat "are given us richly to enjoy."

